# Fusion cytokine IL-2-GMCSF enhances anticancer immune responses through promoting cell–cell interactions

**DOI:** 10.1186/s12967-016-0799-7

**Published:** 2016-02-05

**Authors:** Qian Wen, Wenjing Xiong, Jianchun He, Shimeng Zhang, Xialin Du, Sudong Liu, Juanjuan Wang, Mingqian Zhou, Li Ma

**Affiliations:** Institute of Molecular Immunology, School of Biotechnology, Southern Medical University, #1838, Northern Guangzhou Ave, Guangzhou, 510515 Guangdong Peoples’ Republic of China

**Keywords:** Interleukin-2, Granulocyte–macrophage colony-stimulating factor, Fusion protein, Antitumor, Cell–cell interaction

## Abstract

**Background:**

Potent antitumor responses can be induced through cytokine immunotherapy. Interleukin (IL)-2 and granulocyte–macrophage colony-stimulating factor (GM-CSF) are among the most effective cytokines to induce tumor-specific systemic immune responses and can act synergistically. To overcome the limitations of combined use of these two cytokines, we have constructed an IL2-GMCSF fusion protein and characterized its antitumor effects in this study.

**Methods:**

The expression of IL-2 receptor and GM-CSF receptor of cell lines were detected with quantitative real-time PCR. On this basis, the bioactivities of IL2-GMCSF, especially effects on DC2.4 cells were assayed. Another function of IL2-GMCSF—bridge two types of cells—was assessed by cell contact counting and cytotoxicity assays. The anti-tumor activity in vivo of IL2-GMCSF was evaluated in the melanoma model. The statistical significance among treatment groups were determined by One-Way ANOVA.

**Results:**

The fusion protein IL2-GMCSF maintained the activities of IL-2 and GM-CSF, and could significantly promote DC2.4 cell activities, including phagocytosis, proliferation and cytokine secretion. In addition to the inherent cytokine activity, IL2-GMCSF bridges direct cell–cell interactions and enhances splenocyte killing efficacy against multiple tumor cell lines in vitro. Co-injection of IL2-GMCSF and inactivated B16F10 mouse melanoma cells induced complete immunoprotective responses in about 30 % of mice.

**Conclusion:**

These results suggested that IL2-GMCSF can efficiently regulate immune responses against tumors. Furthermore, as the bridging effect relies on both IL-2R and GM-CSFR and promotes interactions between immune and tumor cells, IL2-GMCSF may be utilized as a useful tool for dissecting specific immune responses for future clinical applications.

## Background

One critical role of the immune system is to maintain host homeostasis. However, tumor cells often establish a local suppressive environment to escape immune surveillance through various mechanisms [1], such as growth repression of immune cells to impair normal immune responses [2], production of immunosuppressive humoral factors to block cytolytic activity of effector cells [3], down-regulation of antigen/MHC complex expression [4], and induction of T cell dysfunction [5] and suppression of T cell activities [6]. Effective antigen presentation and subsequent induction of active T cells in tumor foci are essential to inhibit tumor progression, and among which enhanced cellular crosstalk plays pivotal role.

Immune responses require both direct and indirect cell–cell contacts. The former occurs during dendritic cell (DC) maturation induced by activated natural killer (NK) cells [7], antigen presentation, and the cytolytic activities of cytotoxic T lymphocytes (CTL) against target cells. Direct cell–cell contact not only enhances cellular activation and signal transduction, but also ensures the specificity of immune responses. To enhance direct cell–cell interactions between tumor cells and immune cells, various types of fusion molecules were produced and applied, for example, anti-tumor associated antigen (TAA)-antibodies fused with cytokines [8], TAA linked with a molecular chaperone [9], scFv-scTRAIL [10], OVA-gp100 [11], interleukin-2 (IL-2)-TCR [12], etc. By this way, cell–cell crosstalk is promoted and biological processes are accelerated, exerting enhanced antitumor effects [13]. However, few studies reported the efficacy of a fusion protein composed by two immune molecules.

Cytokine is promising in enhancing indirect cell–cell communication in immune responses [14]. Granulocyte–macrophage colony-stimulating factor (GM-CSF) and IL-2 are among the most powerful cytokines to induce tumor-specific systemic immune responses in experimental models and clinical trials [15, 16]. GM-CSF performs multiple immunoregulatory activities, including promoting differentiation of granulocyte, macrophage, and eosinophil precursor cell [17], as well as stimulation and recruitment of DCs [18]. Meanwhile, GM-CSF also improves the expression of IL-2 receptors on the surface of T cells and is one of the most potent cytokines that exert long-distance antitumor effects [19]. Although IL-2 is mainly produced by T helper cells, functional IL-2 in DCs is still transiently upregulated soon after encountering bacteria, which is critical for DC-mediated activation of T cells [20]. IL-2 is important in tumor exclusion [19] through stimulating effector cells such as CTLs, NK cells, and macrophages [21]. Lymphokine-activated killer (LAK) cells [22] and tumor-infiltrating lymphocytes (TILs) induced by high doses of IL-2 [23] in vitro can infiltrate tumors to destroy them [24], even NK-resistant tumor cells [25]. Moreover, IL-2 contributes to the maintenance of T-cell homeostasis by promoting activation-induced cell death of effector T cells during the late stage of antigen-specific T-cell responses [26].

IL-2 and GM-CSF can not only stimulate the proliferation and cytotoxicity of TILs in the presence of tumor cells [27] but also promote the activation and cytotoxicity of monocytes to attack melanoma in vitro [28, 29] and prolong the survival of polymorphonuclear neutrophils [30]. The amount of activated immune cells in peripheral blood correlates with the survival rate of patients [31], thus the combined applications of IL-2 and GM-CSF were regarded as a promising strategy for cancer immunotherapy. Shinichiro et al. used IL-2 and GM-CSF to culture the α-GalCer-pulsed peripheral blood mononuclear cells and conducted the phase I and I-II studies in patients with non-small cell lung cancer. The results showed that the treatment was safe and antitumor immune responses depending on NKT cell were successfully elicited, which prolonged median survival time [32]. However, the substantial difference in the half-life in vivo and bioactivities of the two cytokines makes the results of their combinatory application unpredictable [33].

To achieve predictable therapeutic effects with combined use of the two potent immunocompetent cytokines, we constructed a IL2-GMCSF fusion cytokine [34]. Such a fusion cytokine has been reported to enhance anti-tumor immune responses and NK cell activities. However, the effect of the fusion cytokine on DC activity has not been explored. In this study, we studied the role of this fusion protein in regulating DC activity, and showed that it functions not only keep the immune activities of both cytokines but also promote direct cell–cell interactions through acting as a bridge to bring different types of cells in close proximity by direct binding with their cytokine receptors respectively which will improve intercellular communications and in turn enhance immune responses.

## Methods

### Animals and cells

The 6-8-week-old male BALB/c mice, C57BL/6 and 5-week-old male nude mice were provided by the Center for Laboratory Animal Sciences of Southern Medical University (Guangzhou, China). FDC-P1 cells (ATCC CRL-12103) and WEHI-3 cells (ATCC TIB-68) (Peking Union Medical University, Beijing, China) were maintained in Dulbecco’s modified Eagle’s medium (DMEM. Hyclone Ltd, Logan, UT) containing 10 % fetal bovine serum (FBS. Hyclone). For culture of FDC-P1 cells, 10 % of WEHI-3 cell-conditioned medium (WEHI-3/CM) was supplemented. CTLL-2 cells (ATCC TIB-214), murine A1.1 T cell hybridoma [35] (kindly gift from Dr. Yufang Shi, The Key Laboratory of Stem Cell Biology, Institute of Health Sciences, Shanghai, China), B16F10 cells (ATCC CRL-6475), B16-GMCSF (B16F10 cells stably transfected with mouse gm-csf gene), DC2.4 and RAW264.7 cells were conserved in our laboratory and maintained in RPMI-1640 medium (Hyclone) supplemented with 10 % FBS. All of the five cell lines were derived from C57BL/6 mouse.

### RNA isolation and real-time quantitative RT-PCR

To detect the expression of IL-2 receptor and GM-CSF receptor, total RNA was extracted using Trizol (Life technologies, Carlsbad, CA) and reversely transcribed using the RevertAid First Strand cDNA Synthesis Kit (Fermentas, Life Sciences, Ontario, Canada) after DNase I (Fermentas) treatment. The sequence of primers used were as below: for mIL-2Rα, FP: 5′-GCAACTCCCATGACAAATCG-3′, RP: 5′-CCCGGAATACACTCGTAGTGAA-3′; for mGM-CSFRα, FP: 5′-CGTGCATATCCCCACCGTAATA-3′, RP: 5′-TGAAGGCACGTTGGATTTTATGA-3′; for GAPDH, FP: 5′-GCACGGTCAAGGCTGAGAAC-3′, RP: 5′-GCCTTCTCCATGGTGGTGAA-3′. Real-time quantitative PCR (qRT-PCR) were performed using the iQ™ SYBR^®^ Green Supermix kit (Bio-Rad, Hercules, CA) on Mastercycler ep realplex^4^ (Eppendorf, Hamburg, Germany). Target mRNA quantification was analyzed using the comparative threshold cycle (Ct) method with the software realplex 2.2 (Eppendorf) as described previously [36].

### Cell proliferation assays

Fusion protein IL2-GMCSF was prepared [34] and conserved in our lab. For detection the activity of IL2-GMCSF, CTLL-2 cells or FDC-P1 cells were cultured in the 96-well microplate (Nunc, Thermo Fisher Scientific, Waltham, MA) with serially diluted IL2-GMCSF for 48 h (for IL-2) or 96 h (for GM-CSF). The cell viabilities were detected using the Cell Counting Kit-8 (CCK-8, Dojindo Laboratorise, Tokyo, Japan) according to the manufacturer’s instruction and compared with the standard curves prepared by cells cultured with serially diluted IL-2 or GM-CSF (both from PeproTech Inc., Rocky Hill, NJ).

### Flow cytometry assay

After incubation with or without IL2-GMCSF (2.5 × 10^3^ IU/mL in terms of the activity of GM-CSF in the fusion protein) at 37 °C for 1 h, A1.1, DC2.4 and WEHI-3 cells were stained with His·Tag^®^ mAb (Novagen, EMD Biosciences, Inc., Darmstadt, Germany) at 37 °C for 1 h and FITC-conjugated rabbit anti-mouse IgG (Jackson ImmunoResearch Laboratories Inc., West Grove, PA) at 4 °C for 30 min in turn. And the fluorescence was analyzed by flow cytometry. For analyzing maturation of DC2.4 cells, the following anti-mouse antibodies were used: anti-CD80-FITC, anti-CD86-APC, anti-CD83-PE, anti-MHC class II (I-A/I-E)-PE-Cyanine7. All these fluorescent antibodies and corresponding isotype antibodies were from eBioscience Inc. San Diego, CA.

To assay the phagocytosis ability of DC2.4 cells, cells with different treatments as indicated in the legends (2 × 10^5^ cells/well) were incubated in triplicate with 1 mg/mL of fluorescein isothiocyanate-dextran (FD40, molecular weight 40,000, approx. 45 Angstroms, Sigma) at 37 °C for 15 min. After washes with PBS, the mean fluorescence intensity (MFI) was assayed by flow cytometry.

### DC maturation and activation

B16F10 cells were seeded at the density of 10^6^/mL in 6-well plates (NEST Biotechnology Co.LTD., Wuxi, China) for 24 h. The supernatant was collected following centrifugation to remove cell debris and used as the tumor cell conditioned medium (TCM). IL2-GMCSF or different cytokines was added in the culture of DC2.4 cells incubated with the B16F10 TCM. Cell phagocytosis was assayed 24 h later, and DC proliferation and mature phenotype was assayed 48 h later by flow cytometry.

### In vitro cytotoxicity assays

Cytotoxicity assays were carried out using a DELFIA EuTDA cytotoxicity kit (Perkin-Elmer Life Sciences, Norwalk, CT, USA) according to the manufacturers’ instruction. Eu-labeled other target cells (5 × 10^3^) were co-cultured with DC-CIKs at the indicated E:T ratio in the legend. The signals were collected using the Varioskan Flash reader (Thermo Fisher) and the specific lysis was calculated using the following formula: [(experimental release − spontaneous release)/(maximum release − spontaneous release)] ×100, where the target cells incubated alone indicated the maximum or the spontaneous release with or without complete cytolysis, respectively.

### ELISA

DC2.4 cells (10^6^/well) in 6-well plates were cultured overnight, and then incubated with the different cytokines. The culture supernatant was collected 24 h later to detect the secretion of IL-12 and macrophage-derived chemokine (MDC/CCL22) with the corresponding ELISA kits (BOSTER Bioengineering Co. Ltd., Wuhan, China) as per the instructions of the manufacture. The absorbance was read using the Varioskan Flash reader.

### Western blot analysis

After treatment as indicated in the legends, total cell protein was extracted using RIPA buffer (ShangHai Biocolor BioScience Technology Company, Shanghai, China) containing 1/10 volumn of PhosSTOP Phosphatase inhibitor Cocktail (Roche), and Western blot analysis was performed as previously described [37]. The following primary antibodies were used with 1:2000 dilution: phosphorylated-NF-κB p65 (p-p65; Ser536; 93H1), NF-κB p65 (D14E12) XP^®^ (Cell Signaling Technology, Inc., Beverly, MA), and GAPDH (Zhongshan Goldenbridge Biotechnology Co., Ltd. Beijing, China). Immunocomplexes on PVDF membrane were detected with appropriate horseradish peroxidase–conjugated secondary antibodies (1:2000; Zhongshan Goldenbridge). The membranes were developed with the SuperSignal West Pico Chemiluminescent Substrate (Thermo Scientific) according to manufacturer’s instructions and the pictures were collected using GeneGnome5 (Gene Company, Ltd. Hong Kong, China).

### Combination assays

Two types of cells (5 × 10^5^/type) were labeled with CellTracker™ Red or CellTracker™ Green (Life technologies), respectively, and then gently mixed together in 100 μL of PBS supplemented with serially diluted fusion protein or medium as well as FBS (100 μL/tube). Anti-human ovarian carcinoma/anti-human CD3 bispecific single chain fusion antibody (BHL-I) (preserved in our lab) was used as the unassociated control. In competition experiments, serially diluted GM-CSF or IL-2 was mixed with IL2-GMCSF and coincubated with cells as indicated in the legends. The mixed suspensions were incubated at 37 °C for 10 min and then centrifuged at RT, 600 rpm for 5 min. After removing about 200 μL/tube of the supernatant, the cells were incubated at 4 °C for 2–4 h. Cell combination was observed under a microscope and cell clusters including no less than three cells were counted. The counting was performed by two different observers who were blind to treatment groups.

### In vivo therapy with IL2-GMCSF

The IL2-GMCSF therapy regimen was designed according to previous study [38] with some modification and approved by the Animal Ethics Committee at Southern Medical University. Two kinds of animal models were established, on C57BL/6 mice and nude mice, respectively. Both of two kinds of animals were randomly assigned to three treatment groups (n = 6 per group). To establish the melanoma model, all C57BL/6 mice received the injection of 5 × 10^3^ B16F10 cells in 50 μL of PBS subcutaneously on day 0, while nude mice were received the injection of 3 × 10^7^ B16F10 cells in 50 μL of PBS subcutaneously into both the right and left back flank. The immune therapy was begun on day 3. For preparation of tumor vaccines, on the day of vaccine administration, 1 × 10^5^ B16F10 cells were inactivated by incubation with 50 μg/mL mitomycin C at 37 °C for 30 min. The inactivated tumor cells were then coincubated with 2.5 × 10^3^ IU/mL of IL2-GMCSF (in terms of the activity of GM-CSF. Named as BF group), combination of IL-2 and GM-CSF (named as 2CK group) or PBS (named as PBS group) at 37 °C for 40 min, each in a column of 50 μL. The above vaccines were subcutaneously injected just around the tumors on the right side while all the left side tumors were injected with PBS as controls. To enhance the immune effects, half dose of the tumor vaccines were administered on day 6 and 12. For therapeutic study, the same treatments were carried out after solid tumor was visible. Similar prime-boost strategy was carried out on day 6 and 12 after the first administration of tumor vaccine. Tumor volumes and animal survival were monitored over time.

### Statistical analysis

Data from the cellular experiments are expressed as the mean ± SD. One-Way ANOVA was applied to analyze the statistical significance. Post hoc multiple comparisons were performed using least Significant Difference or Dunnett’s T3 methods. Statistical analysis of survival data from animal experiments was performed using the Life Tables method. The difference was considered to be statistically significant when *P* is below 0.05. All statistical analyses were performed using SPSS statistical software version 16.0 (SPSS, Chicago, IL, USA).

## Results

### Gene expression assessment of receptors for IL-2 and GM-CSF

**Fig. 1:**
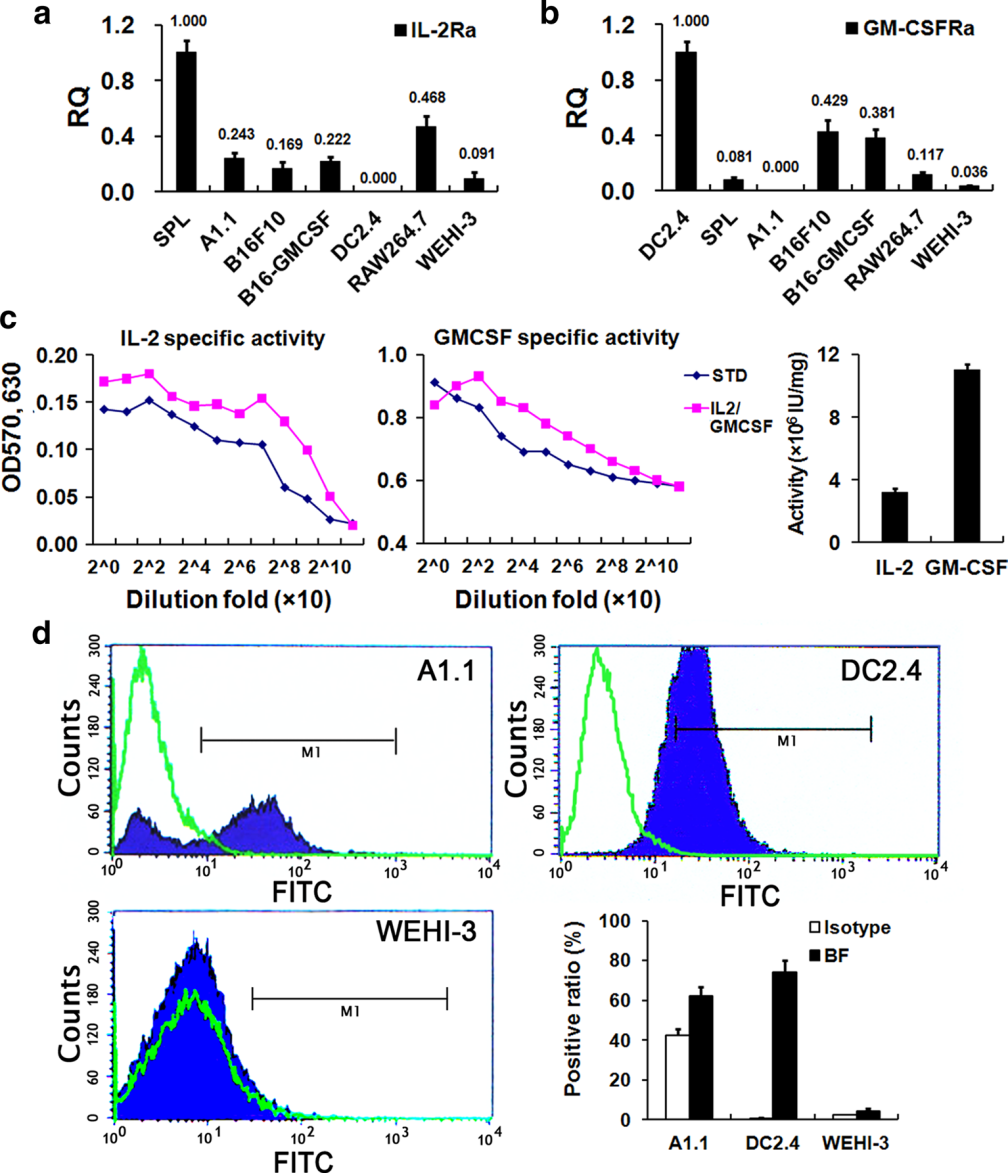
Identification of cell receptor expression and assays of the IL2-GMCSF bioactivity. **a**–**b** qRT-PCR was used to detect the IL-2Rα and GM-CSFRα chain expression in different cell lines; **c** IL2-GMCSF harbored the activities of its component cytokines, as demonstrated by cell proliferation assays of mouse splenocytes for IL-2 acivity and FDC-P1 cells for GM-CSF activity; **d** flow cytometry assays showed that IL2-GMCSF could bind on A1.1 cells (IL-2R^+^) and DC2.4 cells (GM-CSFR^+^), but almost not on WEHI-3 cells which was used as the IL-2R^−^GM-CSFR^−^ control. These experiments were repeated at least three times with similar results

The functional mediator of cytokines is their receptors mainly expressing on the cell surface. To explore the role of IL2-GMCSF in the cell interaction, we firstly evaluated the expression of the IL-2 receptor (IL-2R) and the GM-CSF receptor (GM-CSFR) in different cells using qRT-PCR, including C57BL/6 mouse splenocytes, melanoma cell lines B16F10 and B16-GMCSF, an immature DC cell line DC2.4 [39], a T cell hybridoma A1.1, a macrophage cell line RAW264.7 and a myelomonocytic leukemia cell line WEHI-3. Murine splenocytes and DC2.4 cells were used as the positive controls for IL-2Rα and GM-CSFRα expression, respectively. The results showed that A1.1 cells only expressed IL-2R while DC2.4 cells only expressed GM-CSFR. In contrast, Con A-treated splenocytes expressed both cytokine receptors, consistent with their heterogeneity and indicating the co-existence of lymphocytes and antigen-presenting cells (APCs) such as DCs and macrophages. Unexpectedly, many tumor cell lines, including B16F10, B16-GMCSF and RAW264.7, also expressed both of the two cytokine receptors, just in different levels. By contrast, WEHI-3 cells expressed both receptors in very low levels (Fig. 1a, b).

### Bifunctional activity assessment of IL2-GMCSF

To ensure the fusion cytokine has both IL-2 and GM-CSF activities, the viability of CTLL-2 and FDC-P1 in the presence of serially-diluted IL2-GMCSF was assessed. Results of the WST-8 colorimetric method indicated that the fusion cytokine exerted growth promotion effects on IL-2-dependent splenocytes and GM-CSF-dependent FDC-P1 cells in a dose-dependent manner, which were parallel with both the IL-2 and the GM-CSF standards (Fig. 1c, left and middle panels). The specific activities of this fusion cytokine were 3.6 × 10^6^ IU/mg for IL-2 and 1.1 × 10^7^ IU/mg for GM-CSF respectively, consistent with the results in our previous study [34] (Fig. 1c, right panel). The above assays confirmed this fusion cytokine possessed both of the biological activities of IL-2 and GM-CSF. For convenience of description, the amount of IL2-GMCSF used in subsequent experiments was calculated in terms of the activity of GM-CSF part of this fusion protein.

Subsequently, the binding of IL2-GMCSF with their receptors were examined on IL-2R^+^ A1.1 cells and GM-CSFR^+^ DC2.4 cells, while IL-2R^low^GM-CSFR^low^ WEHI-3 cells were used as the negative control. Indirect immunofluorescence staining indicated that IL2-GMCSF significantly enhances the fluorescence-positive ratio both for A1.1 cells and DC2.4 cells (*P* < 0.05). Consistent with the rare expression of both IL-2 and GM-CSF receptors, the binding activity of IL2-GMCSF to the surface of WEHI-3 cells was at a very low level (Fig. 1d).

### Enhancement of DC2.4 cell activities enhanced by IL2-GMCSF

**Fig. 2:**
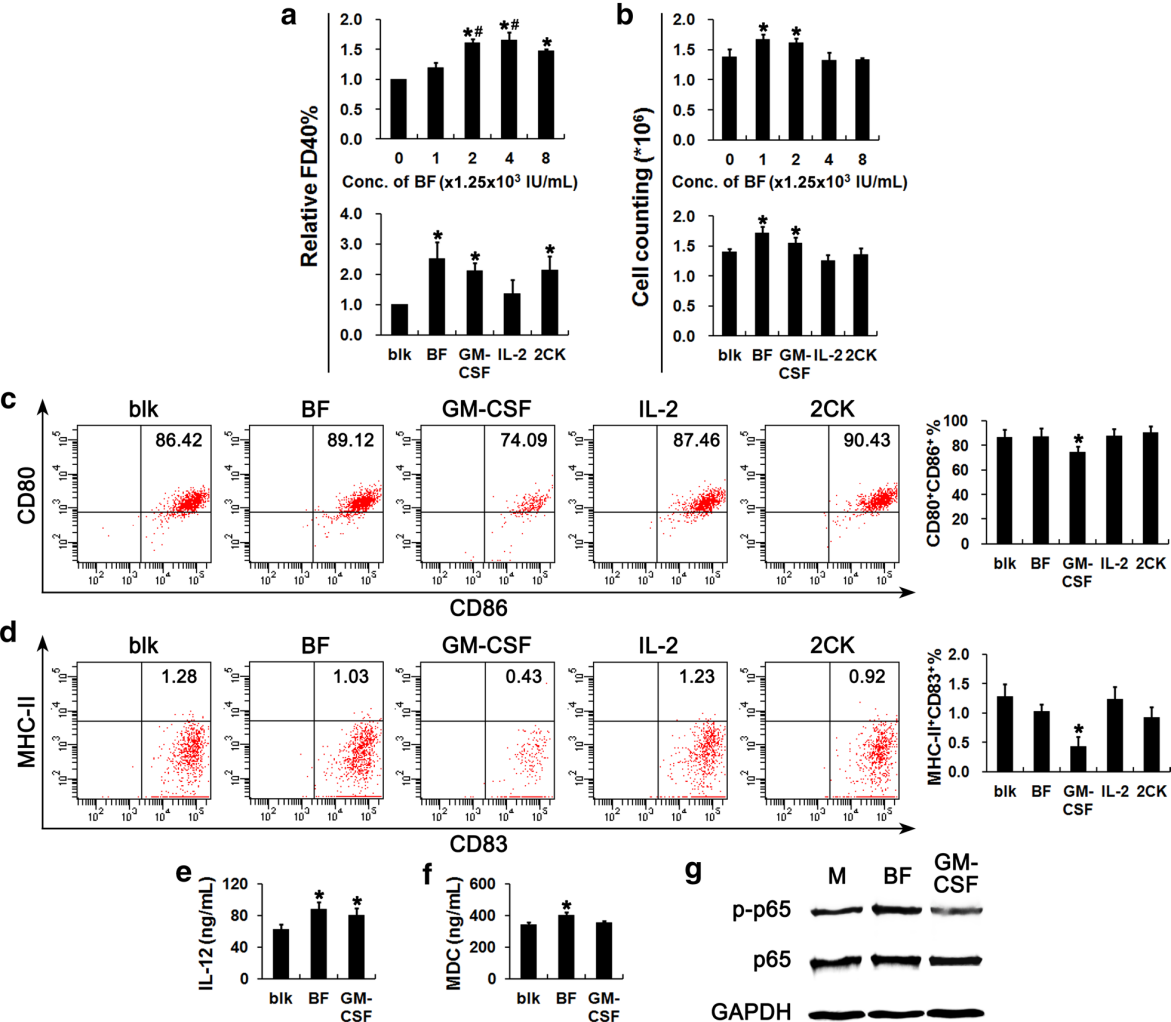
Effects of IL2-GMCSF on DC 2.4 cell bioactivities. **a** FD40 engulfment and **b** cell proliferation of DC2.4 cells under different dosage of IL2-GMCSF or cytokines; **c**–**d** maturation of DC 2.4 cells with different cytokine treatments were indicated by expression of CD80/CD86 (**e**) and MHC-II/CD83 (**f**) detected by flow cytometry. **e** IL-12 and (F) MDC secretion by DC 2.4 cells treated with IL2-GMCSF or GM-CSF were assayed with ELISA. **g** Western blotting was used to detect the activation of NF-κB p65 subunit in DC2.4 cells treated as above. In comparison of the effects of IL2-GMCSF and the cytokines alone or in combination, IL-2 was used at 820 IU/mL and GM-CSF at 2.5 × 10^3^ IU/mL, corresponding to the activity of the two parts of the fusion protein at 2.5 × 10^3^ IU/mL. blk: blank; BF: IL2-GMCSF fusion protein; 2CK: the combination of GM-CSF and IL-2. *P < 0.05 compared with the control without cytokine treatment. These experiments were repeated at least three times with similar results

GM-CSF has been proven to promote the phagocytic capacity of DCs. In this study, the effects of IL2-GMCSF on the phagocytotic activity of the immature DC2.4 cells were examined by assessing the engulfment of FD40 (FITC-labeled 40-kDa dextran) after incubation of cells with IL2-GMCSF 24 h later. Results detected by flow cytometry indicated that IL2-GMCSF enhanced the engulfment of FD40 by DC2.4 cells in a dose-dependent manner. The peak level of phagocytosis appeared at 9.9 × 10^3^ IU/mL, which was similar with the level at 2.5 × 10^3^ IU/mL of IL2-GMCSF (in terms of the activity of GM-CSF) (*P* < 0.05) (Fig. 2a, upper). Further assays showed that this pro-phagocytic effect at 2.5 × 10^3^ IU/mL of IL2-GMCSF was comparable to that of GM-CSF alone also at 2.5 × 10^3^ IU/mL (Fig. 2a, lower). To make sure whether the synergistic effect of the two cytokines exists, the effects of IL-2 alone at 820 IU/mL which is corresponding to the IL-2 activity in IL2-GMCSF and the combination of the two component cytokines at the concentrations comparable with IL2-GMCSF were also compared. The results showed that IL-2 alone has little effect on the engulfing efficiency of DC2.4 cells. Consistently, the phagocytic efficacy resulting from the combined using of IL-2 and GM-CSF was similar to the fusion cytokine or GM-CSF alone (Fig. 2a, lower). These results indicated that IL2-GMCSF significantly enhances the phagocytosis of DCs, which mainly originated from the GM-CSF activity of the fusion protein.

Besides phagocytosis, GM-CSF has also been demonstrated to improve DC proliferation. As results of cell counting 48 h after incubation showed, IL2-GMCSF enhanced the proliferation of DC 2.4 cells, but in a reverse dose-dependent manner. The peak level of proliferation was observed at 1.25 × 10^3^ IU/mL, which was comparable with that at 2.5 × 10^3^ IU/mL (Fig. 2b, upper). Like observed in phagocytosis assays, effects of 2.5 × 10^3^ IU/mL of IL2-GMCSF on the proliferation of DC 2.4 cells were similar with that of GM-CSF alone (Fig. 2b, lower). And the effects seemed not to involve the activity of IL-2, because IL-2 alone could not promote cell proliferation, and the proliferative effects of IL-2 and GM-CSF combination were comparable with that of GM-CSF alone, although lower than that of IL2-GMCSF (Fig. 2b, lower). Considering the opposite effects of IL2-GMCSF on DC proliferation and phagocytosis, we chose the IL2-GMCSF dose at 2.5 × 10^3^ IU/mL for further study, which exerts nearly most excellent promotion on both of the activities of DCs.

To explore the effects of IL2-GMCSF on DC maturation, the phenotype of DC2.4 cells treated with IL2-GMCSF were evaluated and compared with individual cytokine treatment. FACS assays showed that IL2-GMCSF exerted no significant effects on DC2.4 maturation as indicated by similar ratios of CD80^+^CD86^+^ cells and MHC-II^+^CD83^+^ cells, which is consistent with the enhancement on phagocytosis of DC2.4 cells by IL2-GMCSF. It was noteworthy that GM-CSF alone significantly inhibited DC maturation. Although IL-2 alone could not affect DC cell activities as well as maturation, it could counteract the effect of GM-CSF, no matter used combined or fused with GM-CSF because the level of DC maturation stimulated by IL2-GMCSF was similar with that by combined using of IL-2 and GM-CSF (Fig. 2c, d). Albeit the remarkable difference in DC maturation between IL2-GMCSF and GM-CSF, their capacity in induction of IL-12 secretion was similar (Fig. 2e). In contrast, significantly higher level of MDC secretion induced by IL2-GMCSF than GM-CSF’s performance (*P* < 0.05) was observed (Fig. 2f).

Previously, it has been reported that NF-κB pathway play key roles in activation of DCs [40]. Consistent with above effects in stimulation of DC2.4 cells by IL2-GMCSF and GM-CSF alone, western blotting assays showed that IL2-GMCSF could also activate NF-κB signaling pathway as indicated by phosphorylation of p65 unit (Fig. 2g).

### Enhancement of cell–cell interactions of receptor-positive cells by IL2-GMCSF

**Fig. 3:**
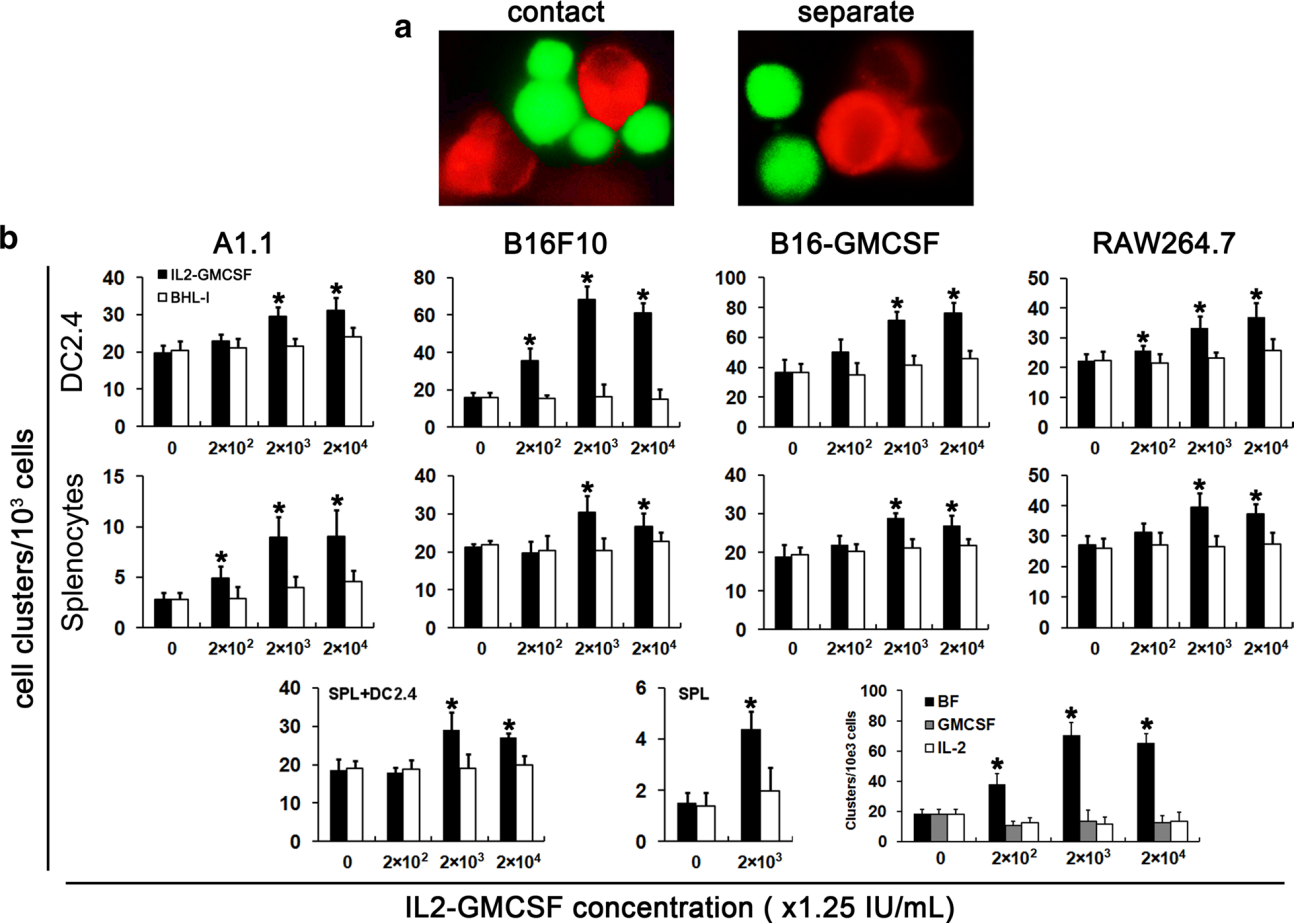
IL2-GMCSF can bring cells into close proximity effectively. Such effects were as demonstrated by the cell cluster counting of several pairs of cell lines incubated with different concentrations of IL2-GMCSF or BHL-I. **a** Typical cell–cell contact (*left*) and separate cells (*right*) were shown by fluorescence microscopy observation after incubation of two types of cells stained with CellTracker™ *Red* or CellTracker™ *Green r*espectively; **b** tumor cell lines including A1.1, B16F10, B16-GMCSF and RAW264.7 were incubated with DC2.4 cells (*upper* panel) or splenocytes (*middle* panel) in the presence different concentrations of IL2-GMCSF. Results of cell binding between DC2.4 cells and splenocytes (*lower* panel, *left*) and among splenocytes (*lower* panel, *middle*) were also shown. GM-CSF and IL-2 were used in the competition experiments to demonstrate the specific combination of IL2-GMCSF with cells (*lower* panel, *right*). The representative result of three repeat experiments with similar results was shown. *P < 0.05 compared with the control without IL2-GMCSF

As IL-2R and/or GM-CSFR are widely expressed among immune cells and tumor cells, we supposed that IL2-GMCSF should be capable of enhancing cell–cell interactions by binding to both receptors and bridging them into close proximity. To test this hypothesis, different types of equal number cells were incubated in pairs with various concentrations of IL2-GMCSF, and individual cell types of coincubated cells are easy to be distinguished under the fluorescence microscope by staining with CellTracker™ Red or CellTracker™ Green respectively. Fusion protein-induced cell–cell interactions were quantified by counting cell contacts between different cell types. To avoid overestimate the contact number of large cell clusters, which contains more than 10 cells of different cell types and one cell in it may possess multiple contacts with different cells (Fig. 3a), the contacts in such large cell cluster were set as 10. The results showed that the presence of IL2-GMCSF could drastically promote the formation of cell clusters and get higher number of contact count which is when compared with the control group in which IL2-GMCSF was replaced by the non-specific fusion antibody. And this enhancement of cell–cell interactions is in a dose-dependent manner (Fig. 3b). The most significant increase of cell contact numbers appeared in DC2.4-involved cell–cell interactions, no matter the co-incubated cells were tumor cells or splenocytes. Relative weak effects were observed in the interactions between splenocytes and tumor cells (Fig. 3b, lower panel, left). In addition, the presence of 2.5 × 10^3^ IU/mL of IL2-GMCSF also significantly promoted the interactions among splenocytes, indicating consistent results with qRT-PCR that there were IL-2R positive lymphocytes and GM-CSFR positive APCs in this mixed cell population (Fig. 3b, lower panel, middle). To demonstrate that the cell contact was mediated by IL2-GMCSF, besides the above non-specific fusion antibody, serially diluted GM-CSF or IL-2 corresponding with the dose of IL2-GMCSF was also added in the coincubation of DC2.4 cells and B16F10 cells, to compete with the combination of the fusion protein and the receptor on the cell surface. The counting results indicated that either GM-CSF or IL-2 could prevent cell cluster formation (Fig. 3b, lower panel, right). In short, these observations demonstrated that IL2-GMCSF can bring different types of IL-2R and GM-CSFR-expressing cells into close proximity, especially interactions involving DCs.

### Enhancement of in vitro cytotoxicity against tumor cells by IL2-GMCSF

**Fig. 4:**
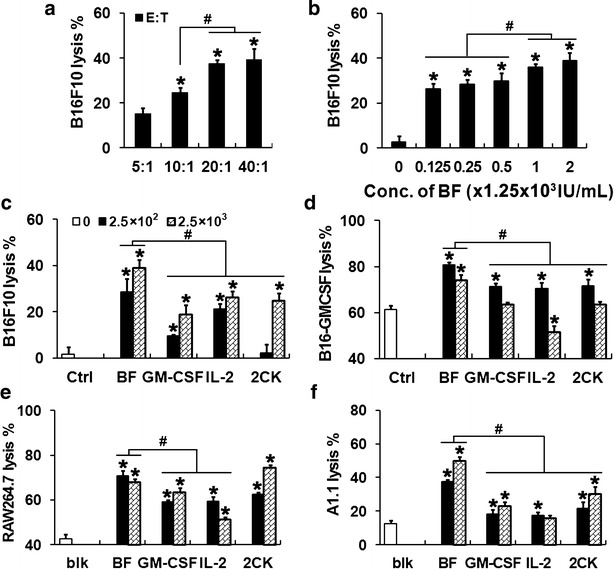
IL2-GMCSF enhanced cytotoxicity in vitro. Cytotoxicity was measured using the WST-8 method to detect the viability of remained target cells. **a**–**b** Effects of E:T ratio of splenoctyes and B16F10 cells (**a**) and IL2-GMCSF dosages (**b**) were determined firstly. Accordingly, cytotoxicity of splenocytes against B16F10 cells (**c**), B16F10-GMCSF (**d**), RAW264.7 (**e**) and A1.1 (**f**) at the 20:1 E:T ratio in the presence of various IL2-GMCSF dosages demonstrated the promotion in cytotoxicity by IL2-GMCSF. *P < 0.05 compared with the control without any cytokine. These experiments were repeated at least three times with similar results

To test the effect of IL2-GMCSF in splenocyte cytotoxicity against tumor cells, B16F10 cells were firstly used as the target cell to evaluate the effects of IL2-GMCSF dosages and the ratio of effector cells to target cells (E:T ratio). Cytotoxicity was determined by detecting cell viability of adherent cells remained after 48 h incubation with suspended effector cells. WST-8 assays showed that, in the presence of 2.5 × 10^3^ IU/mL of IL2-GMCSF, the optimum E:T ratio was 20:1. The cytotoxicity against B16F10 cells at this ratio was significantly higher than at 10:1 or 5:1 (*P* < 0.05), but was comparable to that at 40:1 (*P* > 0.05) (Fig. 4a). This E:T ratio was then used in the subsequent cytotoxicity assays. Meanwhile, the splenocytes effectively killed B16F10 cells in an IL2-GMCSF dose-dependent manner at this E:T ratio (Fig. 4b).

Considering the differences in cell characteristics and possibly consequent IL2-GMCSF effects, two concentrations showing significant differences in the cytotoxicity assay against B16F10 cells were applied to other tumor cells (Fig. 4C). Although the dose-dependent manner of IL2-GMCSF in promoting cytotoxicity was not so significant (*P* > 0.05 compared between different doses) as observed in the assay against B16F10 cells, the fusion cytokine significantly promoted cytotoxicity compared with IL-2, GM-CSF or the cytokine combination (*P* < 0.05) (Fig. 4d–f). Stimulation effects of single or combined cytokines varied greatly with their types and concentrations as well as cell types. For example, the cytotoxicity of the splenocytes was not significantly promoted by the higher concentration of GM-CSF or combined cytokines, and even inhibited in the presence of 820 IU/mL of IL-2 (Fig. 4c, d). Therefore, IL2-GMCSF greatly enhanced the anti-tumor effects of splenocytes compared to either IL-2/GM-CSF alone or their combination.

### Enhancements of in vivo killing efficacy against melanoma by IL2-GMCSF

To evaluate whether IL2-GMCSF could also efficiently function against tumor in vivo, B16F10 melanoma which is a refractory and common cancer was used. Animals were inoculated subcutaneously with B16F10 cells and treated with tumor cell vaccines 3 days later. The tumor cell vaccines were composed of mitomycin C-inactivated B16F10 cells incubated with IL2-GMCSF (BF group), the above two individual cytokines (2CK group) or without any cytokines (PBS group).

**Fig. 5:**
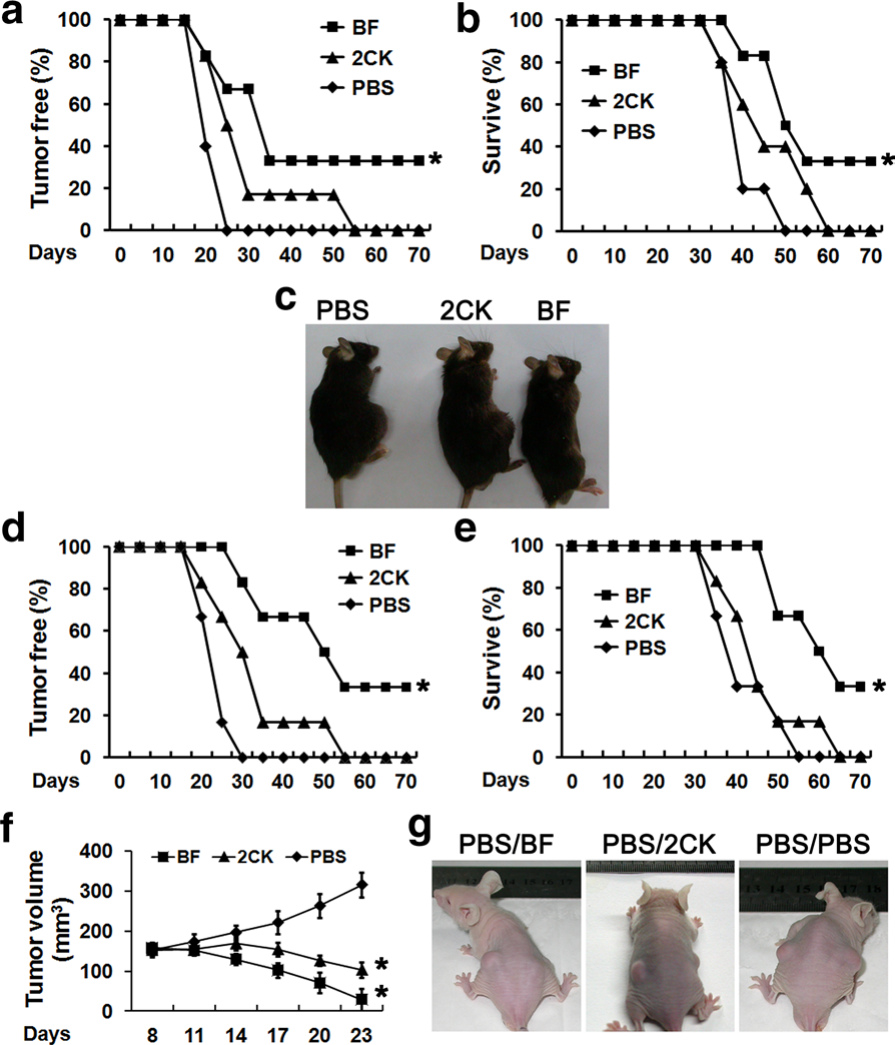
Antitumor activity of IL2-GMCSF in vivo. The percent of tumor-free mice (**a**), surviving mice (**b**) at different time points after treatment of C57BL/6 mice was assessed. The tumor was observed in the C57BL/6 mouse melanoma model after treatment with tumor cell vaccines or PBS (**c**). Furthermore, the percent of tumor-free mice (**d**), surviving mice (**e**) and the tumor volume (**f**) at different time points after treatment of nude mice was assessed. The tumor was observed in the nude mouse melanoma model after treatment (**g**). These experiments were repeated three times with similar results. *P < 0.05 compared with the PBS control group

In C57BL/6 mice, apparent tumor formation was observed in all groups about 20 days after inoculation with B16F10 cells. Although the disease initiation was similar among these three groups, the development of disease was greatly different. The tumor formation was observed quickly in all mice in PBS group, while until 55 days after inoculation was observed in all mice in the 2CK group. In contrast, at the end of observation period, there were still tumor-free mice in the BF group, indicating the complete protection effects (i.e. no tumor formation after inoculation). Meanwhile, the BF group significantly prolonged the survival time compared with the 2CK group and the PBS group (*P* < 0.05) (Fig. 5a–c).

In nude mice, also about 20 days after tumor cell inoculation, tumor formation could be observed in most of groups except the BF group, in which the disease initiation had been delayed for about 5 days. The tumor formation in both the BF group and the 2CK group was significantly later than in the PBS group (*P* < 0.05). The most remarkable protective responses were observed in the BF group (*P* < 0.05) among which 32.6 % of treated animals were completely protected. Similarly, the BF group also prolonged the survival time of nude mice significantly (*P* < 0.05) (Fig. 5d, e).

Above assays were designed and performed to detect the prophylactic function of IL2-GMCSF. In order to demonstrate its therapeutic function, the same tumor vaccines were used to treat the visible solid tumor (about 8 days after inoculation of tumor cells). Notably, decrease in tumor volume was observed in both the BF group and the 2CK group, and the most significant regression appeared in the BF group (Fig. 5f, g). Together, IL2-GMCSF fusion protein displayed strong anti-tumor effects in melanoma animal model.

## Discussion

The immune system functions against tumor progression with complicated mechanisms, which involve cell–cell interactions either through direct contact or indirect communication by cytokines. In this study, we firstly examined the effects of the fusion cytokine IL2-GMCSF on DC 2.4 cell activities in normal and immune-inhibitive conditions, and then demonstrated that IL2-GMCSF acts as a molecular linker which can enhance cell–cell interactions to promote antitumor effects. When functioning as the molecular linker, IL2-GMCSF could play different roles in various conditions. On one hand, IL2-GMCSF can act as an immune enhancer by bringing DCs and other cells in close proximity to enhance the antigen-capturing and presentation efficiency when bound to tumor cells or bound to effector cells, respectively. On the other hand, IL2-GMCSF can also act as a cytotoxic enhancer by pulling effector T cells and tumor cells close to promote specific immune killing.

Two observations may explain the basis of IL2-GMCSF efficacy. First, cellular expression of IL-2R and GM-CSFR widely exist respectively as demonstrated in this study. In particular, almost all tumor cells detected in this study express both the IL-2 and/or GM-CSF receptors. Second, the fusion cytokine retains the activities of both cytokines, including binding to its receptors on cell surface, promoting proliferation of growth factor-dependent cells and enhancing DC activities such as phagocytosis, proliferation and cytokine secretion. DCs are central to immune function, as they participate in the initiation of both innate and adaptive immunity [41] as well as regulatory T cell differentiation [42]. Low number or impaired maturation of DCs will result in greatly compromised antitumor responses [31]. The vital role of DCs in immune responses demonstrates the importance of GM-CSF in immune responses. However, in this study, the results showed that GM-CSF alone inhibited DC maturation. Unexpectedly, the existence of IL-2 could counteract the effect of GM-CSF, no matter used singly or fused with GM-CSF. Because no IL-2 receptor was detected in DC2.4 cells, the mechanism of the counteraction is still unknown. The DC cell line used in this study, DC2.4, represents immature DCs [39] that can develop to mature state after antigen capture and present antigens to effector T cells. Generally, DC2.4 cells are thought to possess the immune activities of natural DCs in vivo.

MDC is a strong chemokine secreted by DCs and macrophages. MDC can recruit large amount of DCs to induce strong anti-tumor immune response. Cao et al. transfected MDC gene into mice and found that, MDC could attract DCs to the tumor loci through inducing IL-4 secretion. Strong antigen-specific immune responses followed, accompanied with obvious repression of tumor [43]. In this study, IL2-GMCSF was demonstrated to own the activity to induce high levels of MDC secretion by immature or mature DC2.4 cells. It was noteworthy that such enhancement in T cell activator IL-12 was also observed, suggesting that IL2-GMCSF could improve the function of DCs in various aspects. Western blot assays showed enhanced activation of NF-κB signaling pathway in DC2.4 cells treated with IL2-GMCSF under both normal, indicating the higher level of activation of DCs by IL2-GMCSF.

As a mixed cell population, splenocytes contain certain amount of APCs, as shown by the expression of GM-CSFR and self-binding promotion by IL2-GMCSF. The promotion of cell–cell interaction by IL2-GMCSF was stronger between DC2.4 cells and tumor cells than between splenocytes and tumor cells, suggesting that binding of IL2-GMCSF to DC2.4 cells may be critical to initiate specific immune response. During cytotoxicity responses, IL2-GMCSF performed multiple roles including improved DC phagocytosis, promotion of cell interactions among DCs, tumor cells and effector T cells, as well as enhancement of T cell activation, which effectively promotes killing efficiency. The cytotoxicity induced by IL2-GMCSF corresponded to the promotion of the interaction between DC2.4 cells and splenocytes which further demonstrates the importance of stimulation on DC2.4 cells by IL2-GMCSF. Together, IL2-GMCSF fusion cytokine promote diverse anti-tumor immune activities.

The prognosis for patients with advanced melanoma remains poor [44]. B16F10 is a malignant melanoma cell line expressing IL-2R [45]. In this study, efficient promotion by IL2-GMCSF on immune cell-binding and cytotoxicity against B16F10 cells were demonstrated in vitro. Accordingly, a mouse melanoma model using B16F10 cells were established. The in vivo results demonstrated in both immune competent C57BL/6 mice and nude mice that the tumor vaccine containing IL2-GMCSF and inactivated B16F10 cells (BF group) exerted the most remarkably protective effects in terms of both the tumor-free ratio and the final survival ratio. And tumor regression in nude mice was also observed in the BF group, which is significant than that in the 2CK group. Darrah et al. speculated that IL-2 mainly functions to expand the activated antigen-specific effector T cells in multifunctional T cells [46], while GM-CSF promotes the cytotoxic activity of effector cells in immune responses such as antibody-dependent cell-mediated cytotoxicity [47]. Vaccination of mice with a poorly immunogenic tumor antigen fused to GM-CSF elicited a potent, long-lasting, and specific antitumor response [48]. GM-CSF may also contribute through its APC chemotactic activity [49]. In this study, the IL2-GMCSF fusion cytokine possessing both IL-2 and GM-CSF activities plays distinct roles but both activities contribute to effective anti-tumor responses. Pre-treatment of the inactivated IL-2R^+^ tumor cells with IL2-GMCSF may provide the highest chance for inactivating tumor cells binding with the IL-2 part of IL2-GMCSF. When the cell-fusion cytokine complexes were injected to tumor region, the GM-CSF part of fusion cytokine may interact with DCs in vivo which is consistent with the stronger interaction between DCs and tumor cells mediated by IL2-GMCSF in vitro. Such interaction may tend to activate DCs and enhance the tumor antigen presentation which in turn induce specific anti-tumor cellular immune response at early stage and contribute to the inhibition of tumor cell growth. And treatment with tumor cell vaccine containing BF protected better than tumor cell vaccine prepared with the two cytokines (*P* < 0.05) which demonstrated that BF has advantages over the combinatory application of individual cytokines, which may result from the linker function that can enhance cell–cell interactions. Interestingly, in this study, the in vivo effects were assessed in two types of mice. In the immunocompetent mice, the effects showed the activities of all cytotoxic cells, mainly should be T lymphocytes. In contrast, in nude mice, the main cytotoxic cells should be NK cells, which express IL-2 receptor and exert important in tumor exclusion as demonstrated in previous report [21]. Thus, the results of nude mice actually should reflect the effects of NK cells, maybe as well as other types of cytotoxic cells exiting in nude mice. These results suggested the full competency of IL2-GMCSF to activate anti-tumor cellular immune response.

The biological function of IL2-GMCSF protein has been reported before. IL2-GMCSF functions as a strong anti-tumor factor in mice [33], and significantly activates NK cells in vitro [50]. IL2-GMCSF has been found to activate the JAK/STAT signaling pathway downstream of IL-2 and GM-CSF, possibly through some special way to combine with their receptors on NK surface. These activities strongly activate NK cells, which make up the loss of NK cell function and number due to advanced bulky malignancies in vivo. In the present study, we focused on another activity of IL2-GMCSF, i.e. the bridging effect. This effect can lead to close contact of neighboring cells and in turn promotion of cell–cell interaction. For the great importance of such interaction in immune response, this bridging effect of IL2-GMCSF can significantly promote the anti-tumor immune response shown in this study and other reports [33, 50].

## Conclusion

In summary, this study reported a promising future for IL2-GMCSF fusion cytokine in the induction and enhancement of anti-tumor immune responses which is much more effective than either of the two cytokines IL-2 and GM-CSF alone and could be potentially applied for developing novel regimen of anti-tumor therapy. Moreover, the immune regulatory role of IL2-GMCSF also support this fusion cytokine is a useful tool to study complex immune response mechanisms, including cytokine activities on immune cells and the effect of direct or indirect interactions among immune cells.

## Abbreviations

DC: dendritic cell
NK cells: natural killer cells
CTL: cytotoxic T lymphocytes
TAA: tumor associated antigen
IL-2: interleukin-2
GM-CSF: granulocyte-macrophage colony-stimulating factor
LAK cells: lymphokine-activated killer cells
TILs: tumor-infiltrating lymphocytes
CM: conditioned medium
qRT-PCR: real-time quantitative PCR
Ct: comparative threshold cycle
MFI: mean fluorescence intensity
MDC: macrophage-derived chemokine
p-p65: phosphorylated-NF-κB p65
